# Pessimism and fearfulness in dairy calves

**DOI:** 10.1038/s41598-017-17214-3

**Published:** 2018-01-23

**Authors:** Benjamin Lecorps, Daniel M. Weary, Marina A. G. von Keyserlingk

**Affiliations:** Animal Welfare Program, Faculty of Land and Food Systems, 2357 Main Mall, University of British Columbia, Vancouver, BC V6T 1Z6 Canada

## Abstract

Animals that experience situations likely to induce negative emotions show changes in judgment associated with pessimism. Few studies have focused on whether animals express stable differences in pessimism and whether these differences are related to personality traits. The first aim of this study was to explore if dairy calves are consistent over time in making judgments under ambiguous situations. Our second aim was to determine whether individual differences in judgment bias are related to conventional personality traits assessed using four standardized tests (Open field, Novel object, Human reactivity and Social motivation test). We subjected animals to two sessions of judgment bias and personality trait tests at 25 and 50 d of age. Individual differences in judgment bias were consistent over time with some animals persistently making more pessimistic choices compared to others. Two main dimensions of personality (Fearfulness and Sociability), obtained through principal component analysis, were also highly consistent over time. Pessimism was related to fearfulness, with more fearful calves making more pessimistic judgments. We conclude that dairy calves differ in the way they perceive and react to ambiguity and that this relates to individual differences in fearfulness.

## Introduction

It is now well recognized that emotional states can induce attention, memory and judgment biases^1^. These affect-induced cognitive changes have been observed in many species^2^. Much interest has focused on the use of judgment biases, assessed by exposing animals to ambiguous situations. Response to ambiguity is believed to reflect pessimism or optimism, defined as an increased propensity to form negative or positive expectations, respectively^3^.

Cognitive biases have been extensively studied in relation to animal welfare^4^. For instance, following an experience predicted to elicit negative emotions such as fear^5^, pain^6^ or chronic stress^7^, farm animals showed a negative bias associated with pessimism. In contrast, subjecting farm animals to environmental enrichment reduced pessimistic judgments, and in some cases caused positive judgment biases^8,9^.

Judgment bias is most often considered as a state that is subjected to change according to the mood of the animal, a consequence of the accumulation of shorter-term emotional responses^10^, but there is a growing body of evidence suggesting that animals express stable individual differences in this response^11^. Pessimism and optimism are considered traits in humans^12^, but to our knowledge the consistency of individual differences in pessimism has received little attention in non-human animals other than rats^13^. More pessimistic rats were found to express higher vulnerability to stress-induced anhedonia^13^, to be more sensitive to negative feedbacks^14^, and to express an inflammatory immune profile compared to optimistic animals^15^, suggesting that trait pessimism might favor the development of depressive states. Recently, a study on dolphins has shown that individuals differ consistently over time in pessimism and that more pessimistic animals show fewer affiliative behaviors^16^. Although pessimism and optimism in humans are related to some of the ‘big five’ personality traits^17^ such as neuroticism and extraversion^18^, to date only one study has linked stable individual characteristics to differences in judgment bias in non-human animals, in this case work on pigs^19^.

Different terms are used to describe individual variation in animals, including personality traits^20,21^, temperament^22^, behavioral syndrome^23^ and coping styles^24^. In the current paper, we use the term trait, but regardless, the underlying idea is that behaviors show consistency over time and across contexts. In the case of farm animals, much of the initial work focused on differences in fearfulness or emotional reactivity^25–27^ defined as a “*general susceptibility to react to a variety of potentially threatening situations*”^28^. This work often addressed how fearfulness affects animal welfare^29^ and productivity^30,31^. Recent studies have found consistent differences in other traits such as sociability^32,33^ and aggressiveness^34^, but few studies have examined how individual characteristics may explain the variation in cognitive processes such as judgment biases.

The aims of this study were to explore whether dairy calves: express consistent differences in the way they perceive and react to ambiguous situations (i.e. judgment bias) and whether these differences are associated with more conventionally assessed personality traits. We predicted that calves would consistently differ in the way they reacted to ambiguous situations and that more fearful animals would make more pessimistic judgments.

## Methods

The study was approved by the University of British Columbia’s Animal Care Committee (# A15-0117) and cared for according to the guidelines outlined by the Canadian Council of Animal Care (2009)^35^.

### Animals

Twenty-two females Holstein calves were enrolled. Within 6 h of birth calves (BW 37.8 ± 4.4 kg) were separated from their dam and fed at least 4 L of >50 g/L of IgG colostrum. Calves were kept in individual pens until 5 d of age. On d 6 calves were moved to a 35 m^2^ group pen (n = 9 ± 1 calves/group). Calves had access to 12 L/d of whole pasteurized milk and *ad libitum* access to water, hay and grain. Fresh sawdust was added weekly to the group pen. Animals were kept with the same pen-mates for the entire experiment.

### General procedure

The spatial learning task required for the Judgment Bias Test was completed first (described below). Training required approximately 12 d. Calves were tested for two consecutive days starting at d 25 and again at d 50.

Personality traits were characterized using four tests applied once per day starting at d 30 (Session 1) and then repeated on d 53 (Session 2). The order of the tests was: Open field (OF), Novel Object Test (NOT), Human’s Reactivity test (HRT), and lastly, the Social Motivation test (SMT) which was divided into two parts: 1) a social isolation and, 2) a runway test.

### Judgment bias test

**Figure 1:**
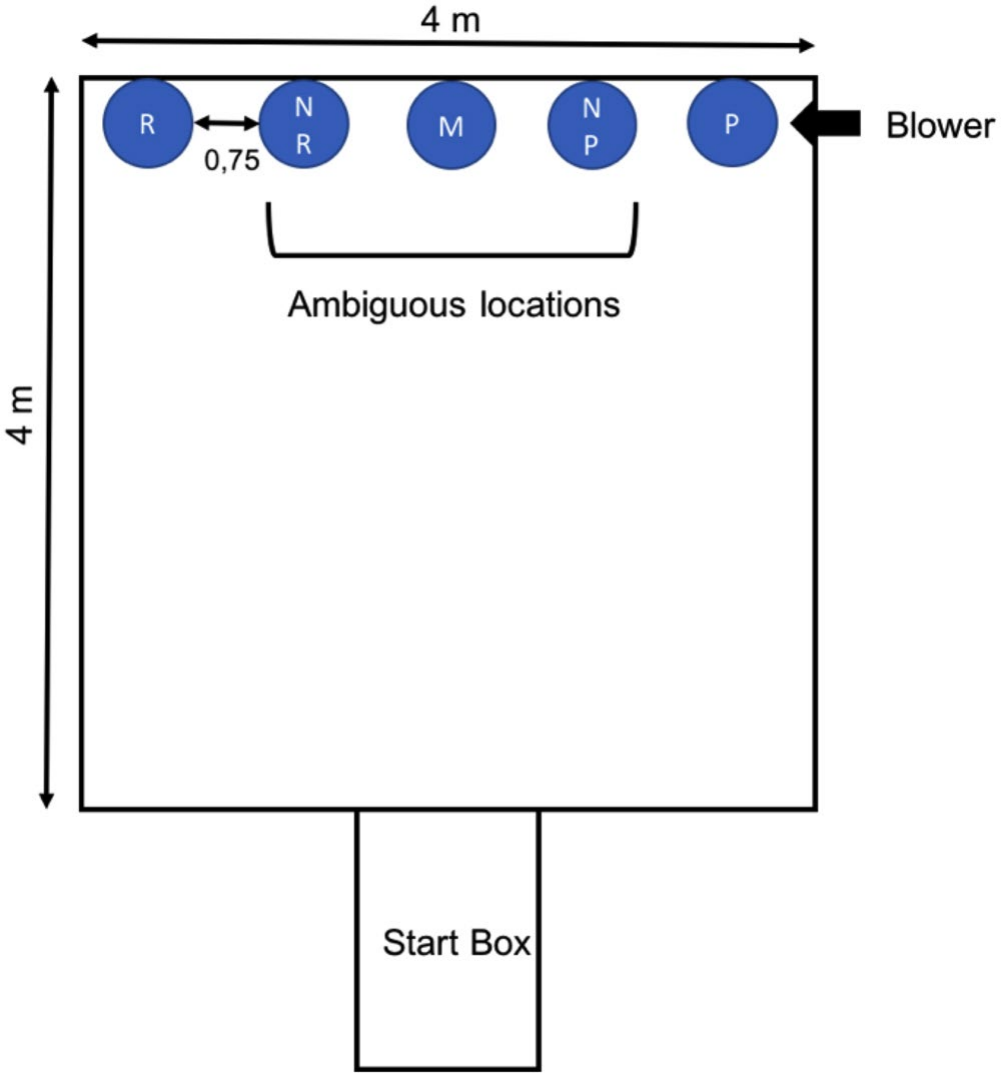
Apparatus designed for training and testing judgment bias. In this example, the rewarded (milk) side was on the right and the punisher (absence of milk + blower) on the left, but training locations were assigned alternately to calves. Once calves met the learning criteria for distinguishing between the rewarded and punished locations, they were presented with ambiguous locations (from left to right: NR: Near Reward, M: Middle, NP: Near Punishment) that were neither rewarded nor punished.

We used a spatial learning task adapted from Destrez *et al*.^5^. Groups of 2 to 3 calves were familiarized with the apparatus (see Fig. 1) for 10 min 1 d before the training phase began but all animals were trained alone. Five consecutive training trials took place each day. During this first 3 d of training, animals were only trained to associate one side of the apparatus (alternately assigned to calves) with a reward (i.e. milk). All animals successfully found the milk holder in less than 30s by the third day of training. Training then introduced negative events: an unrewarded (empty bottle) placed on the opposite side from where the calves had previously received a reward. To facilitate training of the negative event animals that touched the empty bottle received an air-puff in the face as a mild punishment^5,7^. Positive and negative events occurred alternatively during the 5 daily training trials. The last trial consisted of only rewarded events to avoid any negative association with the testing area. On each entry into the testing area, calves were allowed 30 s to approach and touch the milk holder before the response was deemed a ‘no-go’. For calves to be considered as trained they had to approach all positive milk holders (‘go’ response) and never approach the negative milk holder (‘no-go’ response) 10 consecutive times (i.e. 5 trials per day for 2 consecutive days). All animals were trained for 10 d, regardless of when they reached the learning criteria.

Once calves were trained they were tested by placing an empty milk bottle at one of three intermediate (ambiguous) locations: near positive (0.75 m from the positive location), middle (located directly half way (1.5 m) from the positive and negative locations) and near negative (0.75 m from the negative location). On day 1 of testing, animals were exposed first to the positive location (rewarded with milk) and then to the negative one (punished with the blower). Animals were then exposed to ambiguous locations one at a time, beginning with the near positive, followed by the near negative and finishing with the middle location. On day 2 of testing the order was changed to Negative, Positive, Middle, Near negative and Near positive. To ensure that calves remembered the task, we provided two additional days of training on the days before the second test session.

### Personality tests

Except the social motivation test, all tests took place in the same experimental area (a 25 m^2^ test pen) and in all cases animals were tested individually. Animals were gently directed inside the test pen. The 10-min test began once the gate was closed. Behaviors were recorded using a camera (WV-CW504SP, Panasonic, Osaka, Japan) positioned 7 m above the pen. Ethovision XT 10 (Noldus, Netherlands) and BORIS^36^, version 2.05 (www.http://penelope.unito.it/boris) were used for the behavioral analysis.

For the open field test animals entered the experimental pen that was empty and unfamiliar^37^. Distance covered within the pen, time spent exploring and number of vocalizations were recorded. The novel object test involved placing a novel object (a black empty 50 L plastic bucket) in the center of the experimental pen. Latencies to contact the object, duration of contacts, time spent in proximity of the object, total distance covered and number of vocalizations were recorded. The human reactivity test involved an unfamiliar human standing immobile in the middle of the experimental pen. We recorded the latency to contact the human, duration of contacts, time spent at proximity, distance covered and number of vocalizations^38^. The social motivation test is a variant of the runway test used to assess social motivation of dairy cows^32^. This test was done in a familiar alley (25 m × 5 m) that was divided into two distinct spaces (i.e. a holding area and the alleyway) separated by a fence that did not prevent visual contact. For this test, the entire group of calves was moved to the holding pen and given 10 minutes to habituate. One calf was then randomly selected at a time (without replacement) and gently moved to the other end of the alleyway where she was placed in a start box (150 cm × 50 cm) for 5 min. Visual contact with the herd was maintained for the duration of the test. The number of vocalizations was recorded for 5 min, after which the calf was allowed to leave the start box. The time taken to return to within 5 m of the gate separating the rest of the group was measured.

### Statistical analysis

All statistical analyses were made using R version 3.2.1^39^. Inter-observer reliability was evaluated using the intra-class-correlation coefficient (ICC, package ICC) applied to a random selection of 20 videos assessed by two observers. Only duration of exploration of the test environment and duration of contact with the object or the human were measured from video. Observers showed good agreement for both behaviors (duration of exploration: *ICC* = 0.78, *CI*: 0.58–0.98; duration of contact: *ICC* = 0,79, *CI*: 0.55–1,03). One animal was excluded from the study due to an extended period of illness.

### Judgment biases

A linear mixed-effects model (LMM) was calculated with the R package lme4^40^ and P values were extracted by Wald Chi-square tests (type III). The model included the latency to reach the bottle (as the response variable), and tested the effects of distance (in meters) from the rewarded side and session as fixed effects, as well as the interaction between distance and session. Calf was included as a random effect. Model residuals were scrutinized for outliers and normality.

To assess consistency over time, latency to reach each ambiguous location was averaged over the two days. To correct for activity differences, we subtracted the time needed to reach the rewarded bottle from the average latency to reach all ambiguous locations. Measures of latency from all three ambiguous locations were then averaged to get one overall measure of pessimism by session; this was then used to perform linear regression analyses by permutation to test consistency over time (package lmPerm^41^).

### Personality tests

We used averages of the same behaviors expressed in the four different personality tests for each session (e.g. vocalizations in the OF, NO, HRT and SMT were averaged to give only one value per calf per session: Average vocalizations). Only the latency to reach the herd was not averaged with other latencies (latency to contact the object and the human) as the former was used as a proxy for social motivation; the other latencies were used as proxies for fearfulness. A principal component analysis (PCA: package FactoMineR^42^) that summarized correlated variables into principal components, was undertaken using the averages of behaviors expressed in Session 1. Then, to calculate individual PC coordinates for the second session, we used the function predict to perform another PCA on the same space created for the Session 1. This allowed us to obtain comparable PCs for both sessions.

### Relationship between pessimism and personality traits

Measures from Session 1 and 2 were averaged to give an average value for each behavior and a new PCA was performed. Measures included in the PCA were: average number of vocalizations, average distance covered, average time spent in exploration, average latency to contact the object and the human, average time spent at proximity of the object and the human, average time spent in contact with the object and the human and the average latency to reach the herd. Individual coordinates on each of the two principal components were used for further analyses.

Similarly, we averaged the measures of judgment biases arising from Session 1 and 2 (average of latencies to reach all ambiguous locations) to get one individual measure of judgment bias per calf.

The relationship between personality dimensions (individual coordinates in the two components of the PCA) and individual levels of pessimism (mean value of both sessions) was assessed using permutation tests for regression.

## Results

### Judgment biases: Response to ambiguous locations

**Figure 2:**
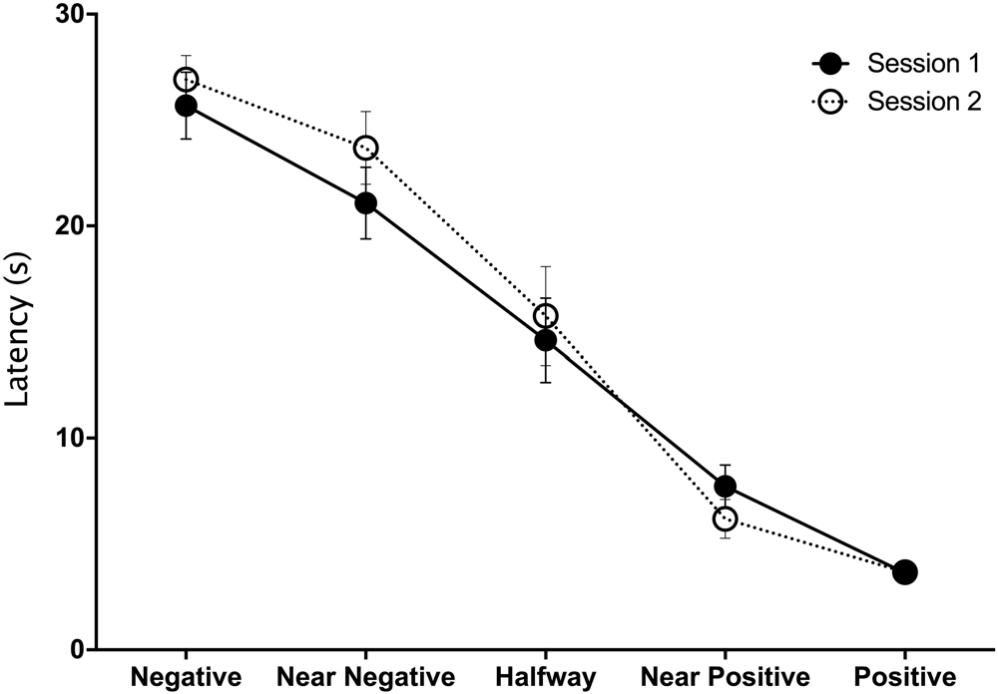
Latency (mean + SEM) to reach the different locations for Session 1 (average of Day 1 and 2) and 2 (average of Day 3 and 4). Calves (n = 21) were trained to associate one side with a reward and the other side with a punishment. Once trained three ambiguous locations were presented between the two previously learnt locations (Near reward, Middle and Near Punishment) and latencies to reach these ambiguous locations were used to assess pessimism.

As expected, the latency to reach the ambiguous bottles was affected by the distance from the reward (Linear regression: Chisq = 192.6, *P* < 0.001), with calves taking longer to approach bottles positioned further from the positive training stimuli (Fig. 2). We found no difference in latency between sessions (Chisq = 0.78, *P* = 0.38). We also found no evidence of an interaction between session and distance (Chisq = 1.7, *P* = 0.19), suggesting that repetition did not affect the way calves responded to the different locations.

**Figure 3:**
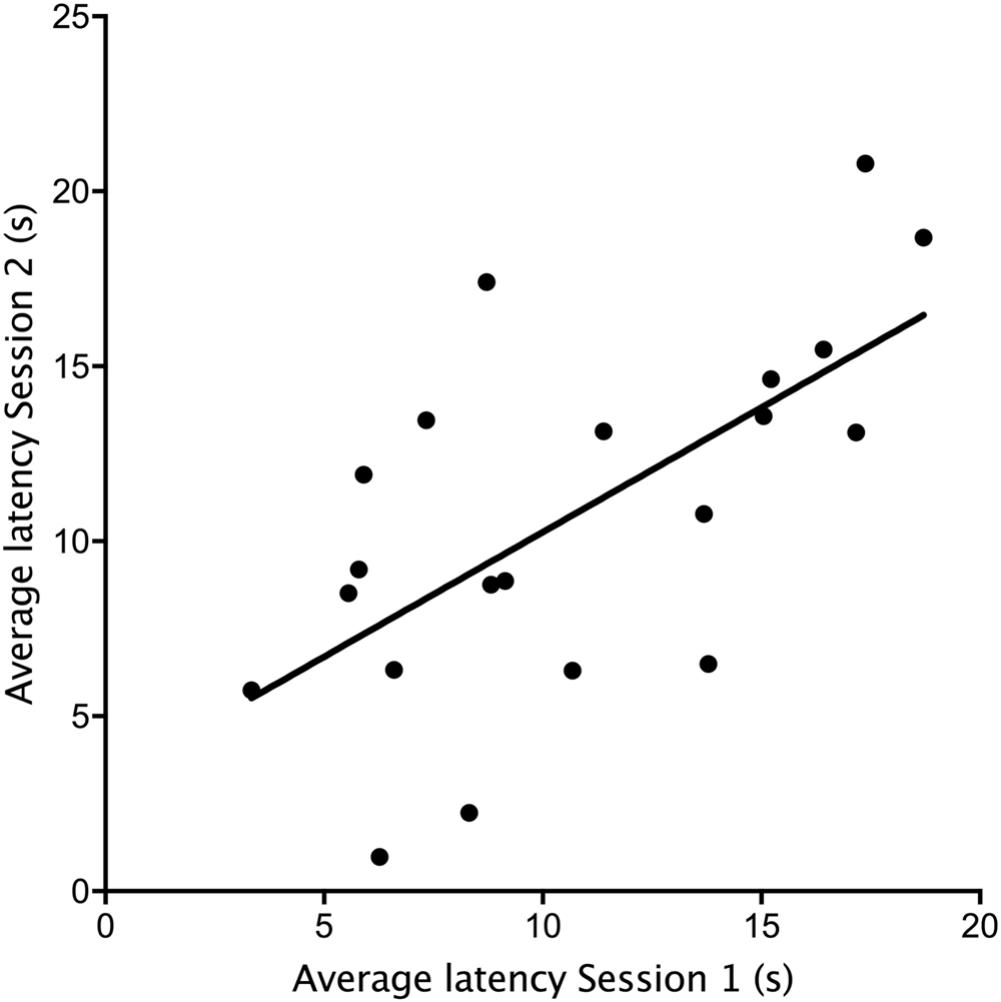
Consistency in the latency to approach ambiguous locations in Session 1 versus Session 2; each point shows the average for a calf based on the latency to approach all three ambiguous locations on both test days within a session (n = 21). The measures were corrected for activity by subtracting the time taken to reach the ambiguous bottles from the time taken to reach the rewarded one.

To assess consistency in judgment bias over time, we averaged the latencies to reach all ambiguous locations for each session. This average latency to reach the three ambiguous locations was consistent over time (Linear regression by permutation: *R*^2^ = 0.41 *β* = 0.57, *P* = 0.002, see Fig. 3).

### Personality traits

**Figure 4:**
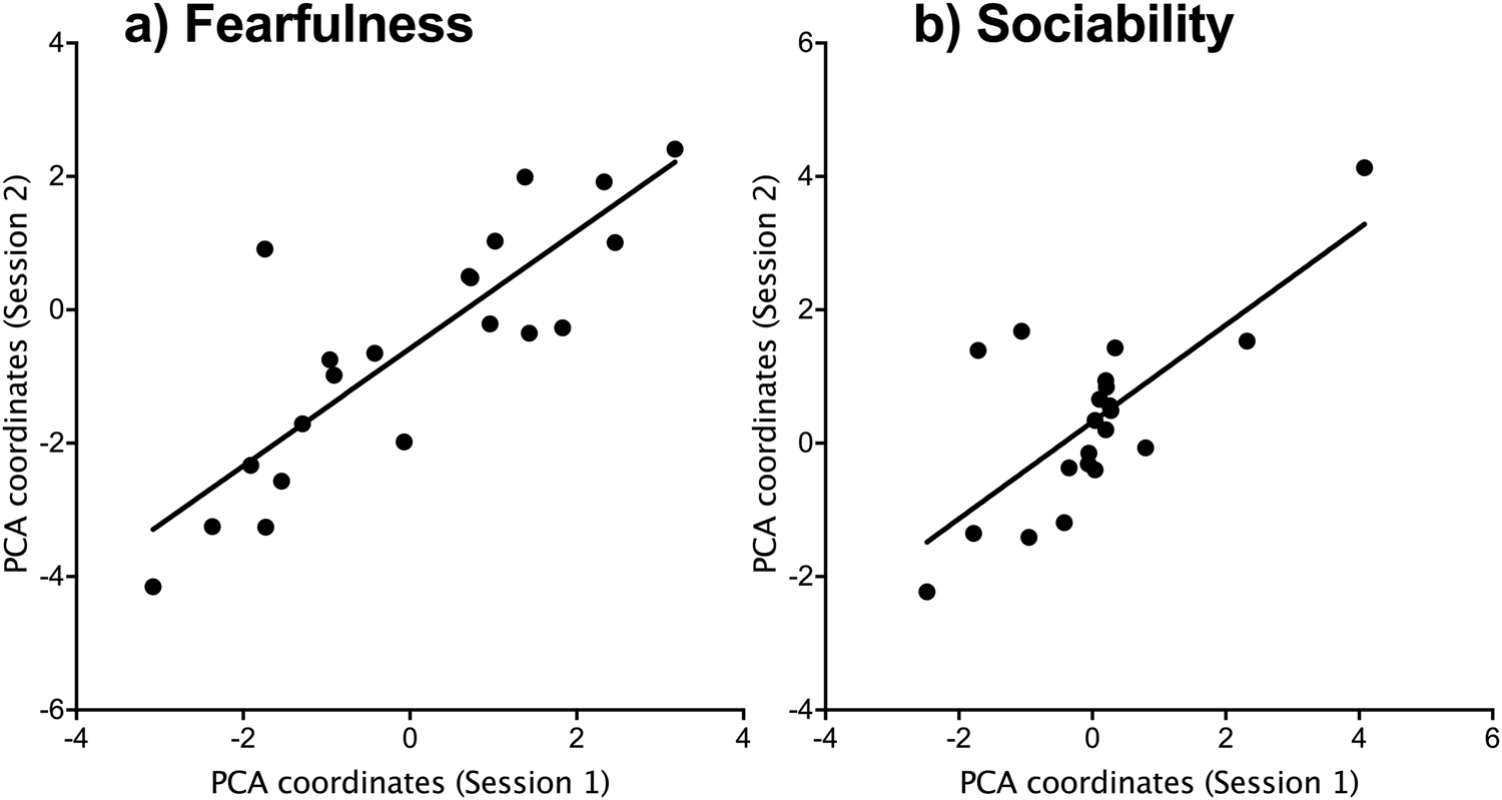
Consistency over time for (**a**) Fearfulness and (**b**) Sociability dimensions. Principal component analyses were performed using averages of similar behaviors expressed in the four tests. Individual coordinates for Session 2 were calculated using the function *predict.PCA* that keeps the same space created for Session 1. Individual coordinates on each component of each PCA were used (n = 21 calves). More positive values mean that calves were either more fearful (slower to make contact with the novel object and the unfamiliar human) or more sociable (high number of vocalizations and short latency to reach the herd).

Averages of similar behaviors expressed in the four different tests (Open Field, Novel Object, Human Reactivity, Social Motivation) were calculated and then used for further analysis. The Principal Component Analysis (PCA) performed for Session 1 revealed two principal components with eigenvalues ≥1. The first component explained 42.6% of the variation and the second component explained 25.6%. The first principal component was primarily explained by the average latency to contact (r = 0.91), the average time spent at proximity (r = −0.94) and the average time spent in contact with the human and the novel object (r = −0.86). The second component was mainly explained by the average number of vocalizations (r = 0.82) and by the latency to reach the herd during the social motivation test (r = −0.66). According to the variable loadings, we labeled the first dimension “Fearfulness” and the second “Sociability”. PCA coordinates have been calculated using the same space for Session 2 and individual PCA coordinates were used to assess the relationship across the two test sessions: Fearfulness (Linear regression by permutation: *R*^2^ = 0.70, *β* = 0.80, *P* < 0.0001; Fig. 4a) and Sociability (*R*^2^ = 0. 53, *β* = 0.73, *P* < 0.001; Fig. 4b) were both consistent across time.

### Relationship between personality traits and individual measures of judgment bias

**Figure 5:**
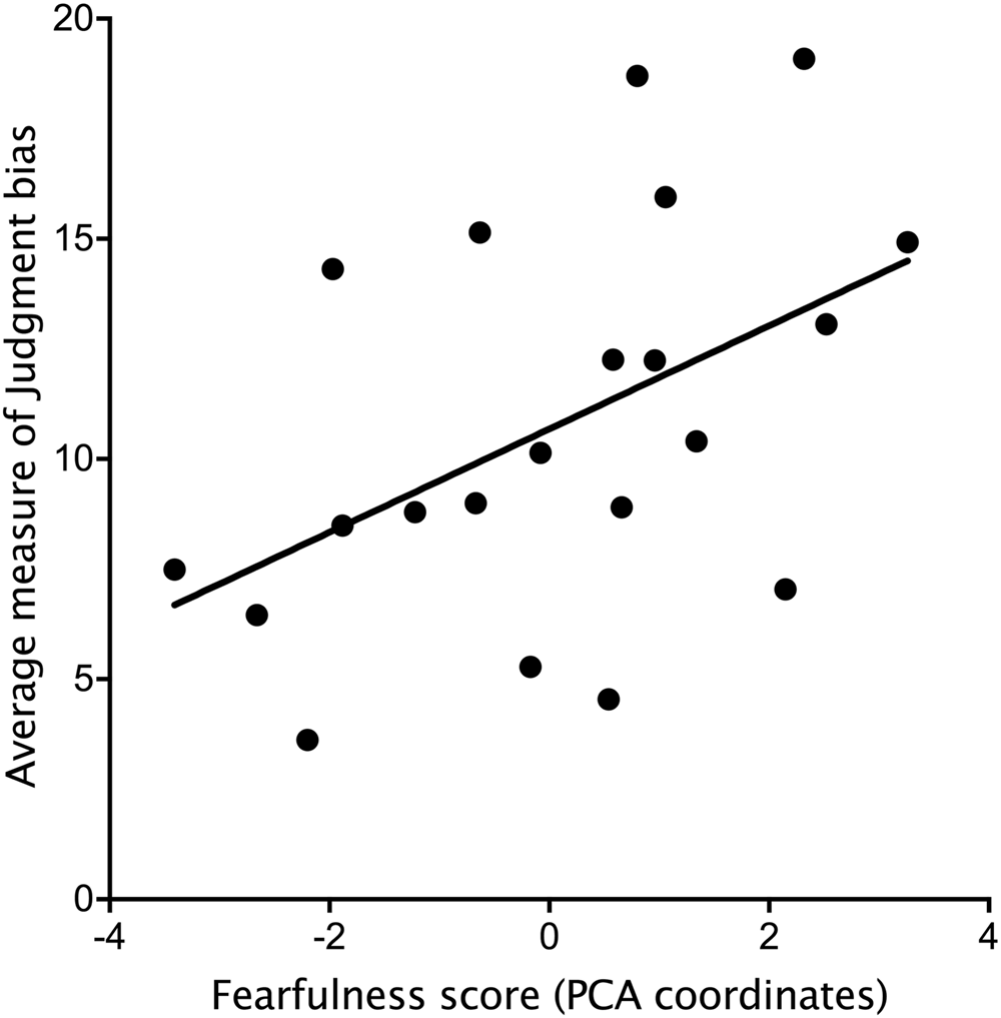
Relationship between fearfulness score determined from the Principal Component Analysis (PCA) and judgment bias. Animals considered more fearful were more reluctant to contact the novel object and the unfamiliar human and had higher positive values on the fearfulness score. The average of judgment biases was obtained by averaging latencies to reach all three ambiguous locations during Session 1 and 2 (n = 21 calves).

A new PCA was performed using averages of the behaviors performed during the personality tests across Session 1 and 2. Individual coordinates on the two first dimensions (with eigenvalues >1) were used to assess the relationship with judgment biases. According to the variable loadings, we again termed these “Fearfulness” and “Sociability” (see Table 1). Fearfulness showed a positive relationship with the average latency to reach the ambiguous locations (Linear regression by permutation: *R*^2^ = 0.24, *β* = 1.21, *P* = 0.023; Fig. 5), indicating that animals that made more pessimistic judgments were also the most fearful (i.e. more reluctant to make contact with the novel object and the human). There was no relationship between sociability and judgment bias (*R*^2^ = 0.06, *P* = 0.29).

**Table 1:** Loadings for the Principal Component Analysis performed on averages of behaviors expressed over the two sessions.

Variables	Dimension 1	Dimension 2
Average_Vocalizations	−0.05	**0.79**
Average_Latency (NO + HR)	**0.87**	0.18
Average_Latency to reach the herd (ST)	−0.12	**−0.76**
Average_Exploration	0.62	−0.63
Average_Contact	**−0.90**	−0.22
Average_Distance	−0.49	0.19
Average_Proximity	**−0.97**	−0.08
Eigenvalue	3.14	1.72
Variance explained (%)	44.8	24.6
Cumulative variance explained (%)	44.8	69.4

## Discussion

The results of this study demonstrate that dairy calves are individually consistent in judgments when confronted with ambiguous situations, and that these individual differences are related to fearfulness as assessed using more conventional personality tests. Two main personality traits emerged from our analysis: Fearfulness and Sociability. Both were consistent over time, but we found no evidence that judgment bias was related to sociability.

Spatial learning tasks have been used to assess judgment bias in animals, but to the best of our knowledge this is the first study to use spatial learning to explore judgment bias in calves. Like many recent studies^5,7,10^, our results show that the latency to explore the probes increased when the distance between the probe and the rewarded side increased. Contrary to some studies^7,43^ we found no evidences of habituation across multiple tests, but in the current study calves were exposed to each probe just twice within each session.

Our results indicate that calves differ in their baseline levels of pessimism and optimism, and do so in a consistent way over a period of 3 weeks. To our knowledge, this is the first time that consistency in judgment bias over time has been demonstrated in farm animals. As recently discussed by Faustino *et al*.^11^, pessimism could be considered as both a state (i.e. changes according to the emotional context) and as a trait with some animals persistently judging ambiguous situations as potentially “negative” or “positive”. Judgment biases occur when humans and animals are subjected to mood changes^1^. Stability in individual levels of judgment bias (referred to here as the trait Pessimism) could be explained by relative time stability in mood state.

These findings have implications for animal welfare, as more pessimistic animals may fail to seize opportunities that are commonly assumed to provide good welfare (e.g. environmental enrichment). For instance, work on rodents has shown that pessimistic rats were less motivated to gain access to a reward^44^. Most importantly, animals that are inherently pessimistic may struggle when faced with emotional challenges. As demonstrated in rats^13^ and as well established in humans^45^, trait pessimism increases vulnerability to stressful events and is thought to contribute to the development of depression. In contrast, animals that are more optimistic may suffer when situations do not lead to reward. For instance, optimistic animals might be more sensitive to discrepancies in expectations and thus may be easily frustrated. This argument has been used to explain the link between optimism and loss of immunity when individuals were subjected to persistent uncontrollable situations^46^. Moreover, this variation may explain the inconsistent results found in some cognitive bias studies in animals^47^, and suggests that individual variation should be taken into account when possible.

According to Roelofs *et al*.^48^, an important validation of judgment bias tests in animals is the existence of correlations between judgment bias scores and personality traits. In the current study, calves that made more pessimistic judgments were also more fearful (i.e. more reluctant to contact the novel object and the unfamiliar human). Pessimistic people tend to engage in avoidance coping strategies when faced with challenging situations^45^ and similar results were recently obtained in pigs^19^; animals showing more pessimistic judgments after environmental change were the less proactive ones (i.e. they made fewer contacts with novel objects and were more distressed by social isolation). In addition, rats that were less anxious in unconditioned fear tests (i.e. open-field and elevated plus maze tests) made more optimistic judgments^49^, suggesting a link between emotional reactivity and judgment bias. Collectively these results suggest that individual differences in fearfulness modulate mood-related cognition in animals as is the case in humans where trait pessimism is positively associated with neuroticism and negatively to extraversion^12^.

To our knowledge, this is the first study to investigate individual variation in animals using averages of similar behaviors expressed in four different tests rather than a single measure arising from one test. Recent work in the human personality literature makes use of aggregated measures across context to better represent personality traits and reports high consistency over time^50–52^. Animal studies investigating personality are often based on behaviors expressed in different situations but with similar causes. For instance, the different tests used in this study have been used to elicit fear responses in farm animals^37^, and tests exposed animals to different forms of social deprivation. Therefore, we expected fearfulness and sociability to be the two main traits explaining individual differences in these tests. Please note that we only aggregated behaviors that we had predicted were similarly motivated. For example, given that different underlying motivations are described with regards to the latency to reach the herd (reflecting sociability; see^32^) and the latency to contact the novel object and the human (reflecting fearfulness; see^37^), we did not average these variables. Although, many previous studies have used PCA^53,54^ and found consistent traits, the use of behavioral averages appears to increase the ability to capture individual differences across different contexts as suggested in the human psychology literature^55^. We encourage future studies to use averaged behaviors in conjunction with PCA.

Our results provide support for the idea that animal personality is multidimensional. Koolhaas and Van Reenen^29^ recently argued that personality traits in animals could be described using two (Fearfulness and Sociability) or three (Fearfulness, Coping styles and Sociability) dimensions. Our study confirms the existence of at least two personality dimensions in dairy calves. Our results did not show clear binomial responses generally associated with coping styles^24^, hence we used the term Fearfulness and Sociability to describe the individual traits in this study. As expected from previous studies^53,56^, latency to contact the human and the novel object and time spent in proximity or in contact accounted for the greatest proportion of variation. The novel object and human reactivity test have been frequently used to assess fearfulness of farm animals^38,57,58^ and usually trigger consistent behavioral and physiological response associated with fear^56,59^. These tests have long been considered reliable measures of fearfulness in farm animals^28,37,38,60^.

Vocalizations have been used as a measure of distress and fear in farm animals^61,62^. However, our results illustrate the social influence on vocal behaviors, given that the number of vocalizations loaded on the second dimension of the PCA, along with the latency to reach the herd after isolation (considered the gold standard to assess social motivation in farm animals^32^). This result agrees with previous work indicating that number of vocal responses of dairy cattle are independent of fearfulness when observed during standardized tests^56^. Similar results have also been reported in sheep^63^ and horses^33^ where vocalizations were related to the expression of social behaviors. These results add to the growing evidence showing stable individual differences in sociability in farm animals^32,33,63,64^.

## Conclusion

Dairy calves show persistent differences in pessimism and in the more traditional personality traits of fearfulness and sociability. All three traits are consistent over time, and individual differences in pessimism are related to fearfulness.

## Electronic supplementary material

Supplementary Information
