# Author Correction: Pessimism and fearfulness in dairy calves

**DOI:** 10.1038/s41598-018-32321-5

**Published:** 2018-09-26

**Authors:** Benjamin Lecorps, Daniel M. Weary, Marina A. G. von Keyserlingk

**Affiliations:** Animal Welfare Program, Faculty of Land and Food Systems, 2357 Main Mall, University of British Columbia, Vancouver, BC V6T 1Z6 Canada

Correction to: *Scientific Reports* 10.1038/s41598-017-17214-3, published online 23 January 2018

In this Article, the legend of Figure 1 is incorrect:

“Apparatus designed for training and testing judgment bias. In this example, the rewarded (milk) side was on the right and the punisher (absence of milk + blower) on the left, but training locations were assigned alternately to calves. Once calves met the learning criteria for distinguishing between the rewarded and punished locations, they were presented with ambiguous locations (from left to right: NR: Near Reward, M: Middle, NP: Near Punishment) that were neither rewarded nor punished.”

should read:

“Apparatus designed for training and testing judgment bias. In this example, the rewarded (milk) side was on the left and the punisher (absence of milk + blower) on the right, but training locations were assigned alternately to calves. Once calves met the learning criteria for distinguishing between the rewarded and punished locations, they were presented with ambiguous locations (from left to right: NR: Near Reward, M: Middle, NP: Near Punishment) that were neither rewarded nor punished.”

